# Electroantennographic response and odorant-binding protein expression alterations induced by host plant volatiles in *Athetis dissimilis* (Hampson)

**DOI:** 10.3389/fphys.2025.1619418

**Published:** 2025-07-09

**Authors:** Yue Qin Song, Tian Si Zhang, Hao Zhan Yuan, Sheng Jie Han, Bo Liao Li

**Affiliations:** ^1^ College of Horticulture and Plant Protection, Henan University of Science and Technology, Luoyang, China; ^2^ Henan Province Engineering Technology Research Center of Green Plant Protection, Luoyang, China; ^3^ Key Laboratory of Applied Ecology of Loess Plateau, College of Life Science, Yan’an University, Yan’an, China

**Keywords:** Athetis dissimilis, plant volatiles, EAG, odorant-binding proteins, molecular docking

## Abstract

Athetis dissimilis is one of the main pests affecting crops, and is currently managed through chemical control methods. Plant volatiles can stimulate insects to feed and lay eggs. The investigation of plant volatiles and the development of effective attractants are essential for sustainable pest control. In this study, we examined plant volatiles using electroantennogram (EAG) analysis, and observed alterations in *OBP* expression in *A. dissimilis*. The EAG results indicated that the EAG responses for *trans*-2-hexenal, *cis*-3-hexen-1-ol, and *trans*-2-hexen-1-ol in male adults were the highest, measuring 1.35 ± 0.02 mV, 1.30 ± 0.10 mV and 1.27 ± 0.18 mV, respectively. This was followed by (*E*, *E*)-2,4-hexadienal (1.01 ± 0.06 mV), octanal (0.69 ± 0.04 mV), hexanal (0.67 ± 0.04 mV), benzothiazole (0.64 ± 0.01 mV), and benzyl acetate (0.61 ± 0.02 mV). The EAG responses of male adults towards the ligands above were generally higher than those of female adults. After fumigation, the expression levels of *AdisOBPs* in both female and male antennae exhibited varying degrees of increase compared with non-fumigated antennae. The expression levels of *AdisOBP9* and *AdisOBP11* in female antennae were significantly higher than those in the paraffin control after fumigation with β-caryophyllene. In addition, after fumigation by β-caryophyllene, the expression levels of *AdisOBP9*, *AdisOBP11*, *AdisOBP24* and *AdisOBP21* in male antennae increased significantly compared with those in the paraffin control. Similarly, after fumigation with benzyl acetate, the expression levels of *AdisOBP9*, *AdisOBP11*, *AdisOBP24*, *AdisOBP21*, *AdisOBP7*, *AdisOBP4* and *AdisOBP50* in male antennae were significantly elevated compared with those in the paraffin control. The number of upregulated genes was greater in male adults than in female adults, which aligns with the results of the EAG test. That is to say, male adults exhibited a stronger response to odor stimulation than female adults. Our findings provide valuable insights into the olfactory mechanisms of insects and contribute to the development of new environmentally friendly pest control methods.

## 1 Introduction

Antennae are important olfactory organs in insects, and their sense of smell is fundamental for their ability to perceive external stimuli and engage in various behaviors, such as food selection, courtship, mating, and spawning. These behaviors play a vital role in the survival and reproduction of insect populations (Metcalf and Kogan, 1987; Bruce et al., 2005; Ha and Smith, 2022). Odorant-binding proteins (OBPs) secreted in the lymphatic fluid of antennal sensilla are directly involved in binding odor molecules, which are then transported to the olfactory receptors on olfactory receptor neurons (ORNs). This process triggers a series of behavioral responses in insects (Pelosi and Maida, 1995; Pei et al., 2024; Tu et al., 2024; Wang et al., 2024). OBPs consist of six α-helices that form a hydrophobic chamber, encapsulating the odor and preventing its degradation (Wogulis et al., 2006; Scott et al., 2009; Li et al., 2019). Understanding the characteristics of OBPs and their interactions with odor molecules is essential for understanding the communication between host plants and insects, as well as for developing effective attractants or repellents. Plants produce significant amounts of volatile compounds during catabolism and anabolism. These compounds play a crucial role in the co-evolution of plants and insects and significantly influence insect behavior (Zhou and Georg, 2021; Pout et al., 2025). For instance, host plants release chemicals, such as hydrocarbons, alcohols, aldehydes, ketones, esters, and organic acids, which characterized by their low molecular weight, diversity, and volatility (Morrissey, 2009). Conducting in-depth research on the mechanisms of chemical perception by insects will aid in deciphering this complex communication system, ultimately leading to more effective strategies for pest control and crop protection.

*Athetis dissimilis* is primarily distributed in China, Japan, Korea, India, the Philippines, and Indonesia, where it poses a threat to various crops including corn, wheat, soybeans, peanuts, and sweet potatoes (Takahashi, 1975; Li et al., 2014). When infesting corn, the larvae of *A. dissimilis* often conceal themselves in the stalk bases of the leftover wheat straw, self-sown wheat seedlings, or weeds, leading to wilting or even the death of the corn plants. This pest frequently coexists with its related species, *A*. *lepigone*, which significantly jeopardizes the safe production of summer corn in China (Li et al., 2014). Numerous studies have focused on the molecular mechanisms of olfaction (Dong et al., 2016; Sun et al., 2016; Song et al., 2021); however, reports on the responses to plant volatiles and the development of plant-derived attractants are limited. In this study, we utilized the electroantennogram (EAG) test and analyzed changes in *OBP* gene expression after stimulation with plant volatiles. Our findings revealed that *A. dissimilis* either attracted or avoided certain odors, and we identified related genes involved in the detection and recognition of plant volatiles. In addition, key amino acid residues were identified using molecular docking analysis.

## 2 Materials and methods

### 2.1 Insect rearing

*Athetis dissimilis* strain was obtained from Shandong Agricultural University in 2017 and was reared in an artificial climate chamber with a temperature of 26°C ± 2°C, a photoperiod of 14 h light and 10 h dark, and a relative humidity of 80% ± 5%. Larvae were fed an artificial diet (including soybean flour, wheat germ flour, bran, yeast powder, vitamin C, sorbic acid, methylparaben, and agar). A 10% honey-water solution was used to supplement the nutrients for the adults. After the mature larvae pupated, distinguished the male from the female, and placed the male and female pupae respectively in the mating cages. Three-day-old unmated female and male adults were used for the EAG and fumigation experiments.

### 2.2 Plant volatiles

According to previous studies on the identification of volatile compounds in maize and wheat (Lan et al., 2022; D'Isita et al., 2024), 61 compounds were selected for the determination of the EAG reaction (Supplementary Table S1). Liquid paraffin was purchased from Shanghai Aladdin Biochemical Technology Co., Ltd. All compounds to be analyzed were diluted with liquid paraffin to a concentration of 1 mol/L and stored at −20°C for future use.

### 2.3 EAG response of A. dissimilis to plant volatiles

The EAG reaction of *A. dissimilis* with volatile compounds in the host plants was assessed using the method described by Li et al., 2022. Adult antennae were excised from the base using ophthalmic tweezers and the tip of the antennal flagellate segments were removed with a sharp blade under an anatomical mirror. The two ends of the antennae were affixed respectively to positive and negative electrodes with conductive glue. The potential signals from the antennae were amplified by an Syntech IDA232 device (Synchronous Technology Co., LTD., Shenzhen, China) and connected to a host computer, where the amplitudes of the antennal potential response were recorded using EagPro V2.0.2 software. A strip of filter paper measuring 3 cm length and 0.5 cm in width was cut and positioned the inner wall of the 200 µL pipette tip. Subsequently, 20 µL of the prepared liquid was applied to the center of the concave strip. To prevent contamination of the odor tube by chemical compounds, the strip must be at least 0.5 cm away from the open of 200 µL pipette tip. The distance from the antenna to the opening of the odor stimulation tube was set to 1 cm. Both the continuous and pulsed stimulated gases for humidification were regulated using a Syntech CS-55 gas generator (Synchronous Technology Co., LTD., Shenzhen, China) with a continuous gas flow rate of 450 mL/min and a pulsed gas flow rate of 120 mL/min. Each compound was stimulated for 0.5 s, with a 60-s interval between stimuli. 6-7 compounds were detected by each antenna and presented in random order. Each adult was tested with only one antenna, and ten antennae were used for each compound. The measurements of liquid paraffin were compared before and after testing for each compound (EAG_CK1_ and EAG_CK2_). The relative value of the EAG reaction was calculated using the following formula:

$$\text{Relative}\;\text{value}=\text{absolute}\;\text{value}\;\text{of}\;\text{treated}\;\text{EAG}\;\text{reaction}-\;\left({\text{EAG}}_{\text{CK}1}+{\text{EAG}}_{\text{CK}2}\right)/2$$

EAG responses concentration-dependent test was conducted using 17 volatile compounds that elicited heightened EAG response in male and female adults. Each compound was successively diluted with liquid paraffin at a 10-fold decreasing rate, resulting in six concentration gradients: 1, 10^−1^, 10^−2^, 10^−3^, and 10^−4^ mol/L. Different concentrations of the same compound were measured, from low to high concentrations. Liquid paraffin served as a control both before and after stimulation with each compound concentration. The concentration-response of a single antenna was measured for each compound and the concentration gradient of each compound was assessed using ten antennae.

### 2.4 Probing AdisOBPs expression by exposure to plant volatiles

Based on the results of the EAG experiment and the Y-tube test (Supplementary Figures S1, S2), two host plant volatiles benzyl acetate and β-caryophyllene, were selected for chemical fumigation tests to assess the expression of the *AdisOBP* genes. 30 male and 30 female adults who had not mated for 3 days after emergence were selected, starved for 24 h and respectively placed in plexiglass cages (35 × 35 × 35 cm). A culture dish was positioned at the center of each cage, with a rubber septum inside the dish, onto which active ingredients 10 mg plant volatiles were applied. Liquid paraffin fumigation and non-fumigation were used as a control. Each group was treated with 4 biological replicates. After 24 h of exposure to this environment, the 60 male and 60 female antennae were respectively removed, immediately placed in liquid nitrogen, and stored at −80°C until further use.

### 2.5 Quantitative real-time PCR analysis

Total RNA was extracted using an RNAiso Plus kit (TaKaRa, Dalian, China). First-strand cDNA was synthesized using PrimeScript RT reagent and gDNA Eraser (TaKaRa, Dalian, China), according to the manufacturer’s instructions. Real-time quantitative PCR was conducted on a Bio-Rad instrument (Bio-Rad Laboratories, Inc., United States) using SYBR® Premix Ex Taq II (TaKaRa, Dalian, China). All *AdisOBPs* and qPCR primer sequences (Supplementary Table S2) were sourced from a previous study ^[19]^. GAPDH served as an internal reference gene to account for variations between samples. Each amplification reaction was carried out in a 20 μL reaction mixture under the following conditions: denaturation at 95°C for 3 min, followed by 40 cycles of 95°C for 10 s and 58°C for 30 s. No template controls were included to detect potential contamination. Three biological replicates were analyzed, and the relative expressions of the *AdisOBPs* were quantified using the −2^−ΔΔCT^ method (Livak and Schmittgen, 2001). Expression was calculated relative to levels of *AdisOBPs* in the female and male antennae of non-fumigation, which were arbitrarily set to 1.

### 2.6 Modeling and molecular docking

Blastp was used to search for protein spatial structure templates in the Protein Data Bank. The Swiss-Model online service (https://swissmodel.expasy.org) was used to enhance the simulation of the three-dimensional structure of the protein (Waterhouse et al., 2018). Molecular docking of AdisOBPs with odor compounds was conducted using the CB-Dock2 online platform (https://cadd.labshare.cn/cb-dock2/index.php) (Liu J.et al., 2022). LigPlot Software (v. 2.2.8) was used for the 2D interaction plotting (Laskowski and Swindells, 2011). Three-dimensional structure illustrations were created using PyMOL software (v. 2.5.0, open source).

### 2.7 Data analysis

SPSS software (version 23.0) was used for statistical analysis of the experiment data. One-way analysis of variance, followed by the Student-Newman-Keuls (S-N-K) multiple comparison, was used to assess the relative value of EAG responses to various plant volatiles, different dosage gradients of the same plant volatiles, and the relative expression levels of *AdisOBPs* after fumigation with different plant volatiles. An independent samples *t-*test (equal variance hypothesis) was conducted to analyze the significant differences in the EAG responses values between male and female insects exposed to the same plant volatiles.

## 3 Results

### 3.1 EAG response to a fixed concentration of host plant volatiles

**FIGURE 1:**
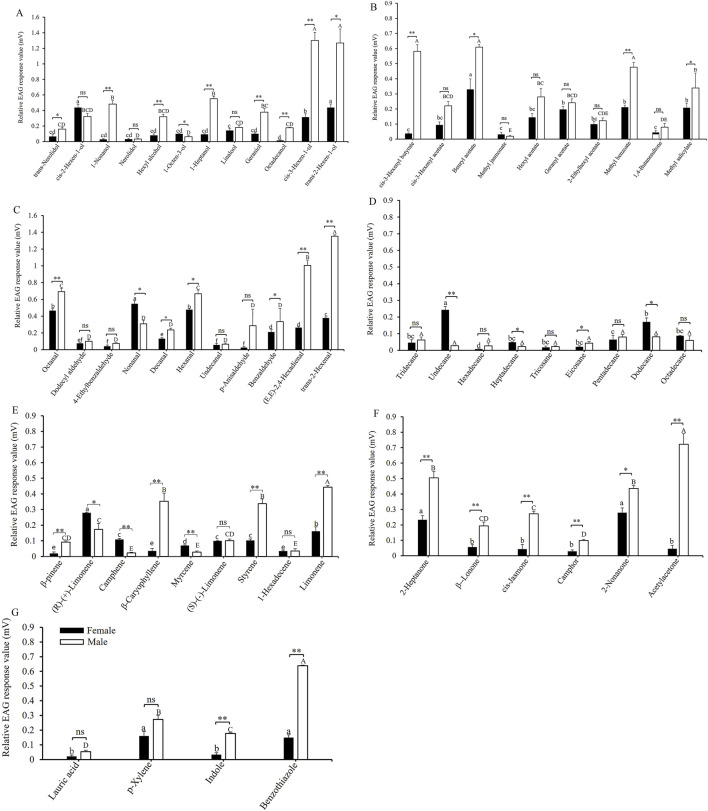
EAG responses of *A*. *dissimilis* adults to 61 host-plant volatile compounds. **(A)** alcohols; **(B)** esters; **(C)** aldehydes; **(D)** alkanes; **(E)** alkanes; **(F)** ketones; **(G)** other compounds. Data are presented as mean ± SE (*N* = 10). Different lowercase and capital letters above bars indicate significant differences (*P* < 0.05) in the EAG response values of female and male adults to different host plant volatile compounds. The single asterisk and double asterisk indicate significant differences (*P* < 0.05) and extremely significant differences (*P* < 0.01), respectively, in the EAG response values between female and male adults for the same volatile compound. *t*-test assuming equal variances (SPSS 23.0).

Figure 1 illustrates the EAG responses of adult insects to 61 host plant volatiles. The relative EAG responses for *cis*-3-hexen-1-ol and *trans*-2-hexen-1-ol in male adults were the highest, measuring 1.30 ± 0.10 mV and 1.27 ± 0.18 mV, respectively, significantly surpassing those of other similar compounds. In contrast, the EAG responses of female adults to alcohols were relatively weak, with the strongest responses observed for *cis*-2-hexen-1-ol and *trans*-2-hexen-1-ol, both at 0.44 ± 0.04 mV. There were significant differences in the responses of male and female adults to the same alcohol substances, such as the EAG responses of male adults to *trans*-2-hexen-1-ol (*t* = 4.445, *df* = 8, *p* = 0.011), *cis*-3-hexen-1-ol (*t* = 8.880, *df* = 8, *p* = 0.001), octadecanol (*t* = 21.112, *df* = 8, *p* < 0.0001), geraniol (*t* = 5.516, *df* = 8, *p* = 0.005), 1-heptanol (*t* = 15.123, *df* = 8, *p* < 0.0001), hexyl alcohol (*t* = 6.914, *df* = 8, *p* = 0.002), 1-nonanol (*t* = 9.784, *df* = 8, *p* = 0.001), and *trans*-nerolidol (*t* = 2.814, *df* = 8, *p* = 0.048) were significantly stronger than that of female adults (Figure 1A). Male adults exhibited the strongest EAG responses to benzyl acetate, *cis*-3-hexenyl butyrate, and methyl benzoate, with values of 0.61 ± 0.02 mV, 0.58 ± 0.05 mV, and 0.48 ± 0.03 mV, respectively. Except for *cis*-3-hexenyl butyrate (*t* = 12.237, *df* = 8, *p* < 0.0001), benzyl acetate (*t* = 3.903, *df* = 8, *p* = 0.017), and methyl benzoate (*t* = 7.616, *df* = 8, *p* = 0.002), there were no significant differences in the EAG responses of male and female adults to the other six ester compounds (Figure 1B). The EAG response to *trans*-2-hexen-1-al (1.35 ± 0.02 mV) was the strongest among male adults, followed by (*E*, *E*)-2,4-hexadienal (1.01 ± 0.06 mV), octanal (0.69 ± 0.04 mV), and hexanal (0.67 ± 0.04 mV). Female adults exhibited a significantly higher EAG response to nonanal (*t* = −4.245, *df* = 8, *p* = 0.013) than male adults, whereas their responses to other volatiles were either significantly lower or not significantly different from those of male adults, such as the EAG responses of male adults to *trans*-2-hexenal (*t* = 45.152, *df* = 8, *p* < 0.0001), (E,E)-2,4-hexadienal (*t* = 11.499, *df* = 8, *p* < 0.0001), hexanal (*t* = 4.019, *df* = 8, *p* = 0.014), and octanal (*t* = 4.880, *df* = 8, *p* = 0.008) were significantly stronger than that of female adults (Figure 1C). The EAG response of female adults to undecane (0.24 ± 0.02 mV) (*t* = −10.372, *df* = 8, *p* < 0.0001) was the highest and was significantly greater than that of male adults. Except for undecane (*t* = −10.372, *df* = 8, *p* < 0.0001) and dodecane (*t* = −3.725, *df* = 8, *p* = 0.020), there were no significant difference in the EAG responses of male and female adults to other alkanes. (Figure 1D). The EAG value for limonene (0.44 ± 0.01 mV) was the highest among male adults, followed by β-caryophyllene (0.35 ± 0.05 mV) and styrene (0.34 ± 0.03 mV). The EAG responses of male adults to limonene (*t* = 9.610, *df* = 8, *p* = 0.001), styrene (*t* = 7.255, *df* = 8, *p* = 0.002), β-caryophyllene (*t* = 5.755, *df* = 8, *p* = 0.005), and β-pinene (*t* = 5.563, *df* = 8, *p* = 0.005) were significantly stronger than that of female adults (Figure 1E). The EAG response of male adults to acetylacetone (0.72 ± 0.07 mV) was the highest, followed by 2-heptanone (0.50 ± 0.04 mV) and 2-nonanone (0.44 ± 0.02 mV). The EAG responses of male adults to all measured ketone compounds were significantly greater than those of female adults, such as the EAG responses of male adults to acetylacetone (*t* = 9.333, *df* = 8, *p* = 0.001), 2-heptanone (*t* = 5.361, *df* = 8, *p* = 0.006), β-lonone (*t* = 4.520, *df* = 8, *p* = 0.011), *cis*-jasmone (*t* = 6.561, *df* = 8, *p* = 0.003), camphor (*t* = 6.058, *df* = 8, *p* = 0.004), and 2-nonanone (*t* = 4.329, *df* = 8, *p* = 0.012) were significantly stronger than that of female adults (Figure 1F). The EAG response of male adults to benzothiazole (0.64 ± 0.01 mV) (*t* = 25.604, *df* = 8, *p* < 0.0001) was the highest and significantly exceeded that of female adults (Figure 1G). Secondly, there was indole (0.178 ± 0.005 mV), and the response of male adults to it (*t* = 10.366, *df* = 8, *p* < 0.0001) is also significantly stronger than that of female adults. Overall, the EAG response of male adults demonstrated greater sensitivity to odors than female adults across all tested compounds in *A. dissimilis*.

### 3.2 EAG response of A. dissimilis to volatile compounds at different concentrations

**FIGURE 2:**
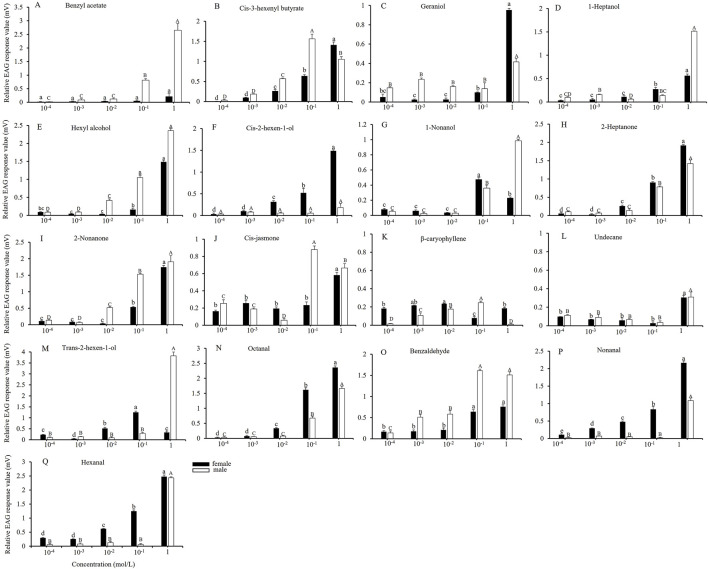
EAG responses of *A*. *dissimilis* adults to different concentrations of 17 volatile compounds Data are presented as mean ± SE (*N* = 10) **(A–Q)**. Different lowercase and capital letters above the bars indicate significant difference in the relative EAG response values of female and male adults with different concentrations of volatile compounds, respectively (S-N-K test groupings, *P* < 0.05).

From the 61 host plant volatilities, 17 compounds were selected to elicit strong EAG responses in both female and male adults, and the EAG responses of the adults to varying concentrations of these compounds were measured. As illustrated in Figure 2, increasing the compound concentration from 10^−4^ to 10^−3^ mol/L did not result in a significant change in the relative EAG response values of female and male adults to the various compounds. At a concentration of 10^−2^ mol/L, the EAG response of male adults to *cis*-3-hexenyl butyrate, hexyl alcohol, 2-nonanone, and β-caryophyllene increased significantly (Figures 2B,E,I,K). In contrast, female adults exhibited significant increases in EAG responses to *cis*-3-hexenyl butyrate, *cis*-2-hexen-1-ol, 2-heptanone, *trans*-2-hexen-1-al, octanal, nonanal and hexanal (Figures 2B,F,H,M,N,P,Q). The EAG response values for male adults to *cis*-3-hexenyl butyrate, *cis*-jasmone, β-caryophyllene and benzaldehyde (Figures 2B,J,K,Q), and for female adults to 1-nonanol and *trans*-2-hexen-1-al reached their maximum values at a concentration of 10^−1^ mol/L (Figures 2G,M). When the concentration was increased to 1 mol/L, the EAG response of both male and female adults to most plant volatiles peaked. Such as male adults responded to benzyl acetate, geraniol, 1-hydroxyheptane, hexyl alcohol, 1-nonanol, 2-heptanone, 2-nonanone, undecane, *trans*-2-hexen-1-al, octanal, nonanal, and hexanal (Figures 2A,C–E,G–I,L–N,P,Q), whereas female adults responded to *cis*-3-hexenyl butyrate, geraniol, 1-hydroxyheptane, hexyl alcohol, *cis*-2-hexen-1-ol, 2-heptanone, 2-nonanone, *cis*-jasmone, undecane, octanal, nonanal and hexanal (Figures 2B–F,H–J,L,N,P,Q).

### 3.3 Plant volatiles induce alterations in AdisOBP expression

**FIGURE 3:**
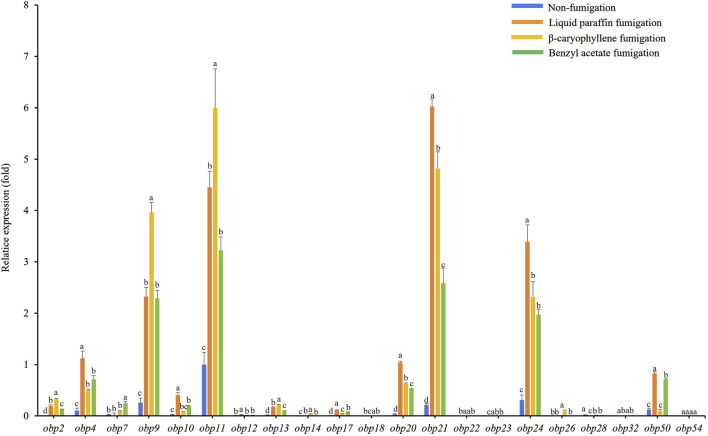
Effect of exposure to plant volatiles on expression levels of *OBP* genes in antennae of *A*. *dissimilis* female adults Data are presented as mean ± SE. Different lowercase letters above the bars indicate significant difference between different treatments of the same gene (*P* < 0.05).

**FIGURE 4:**
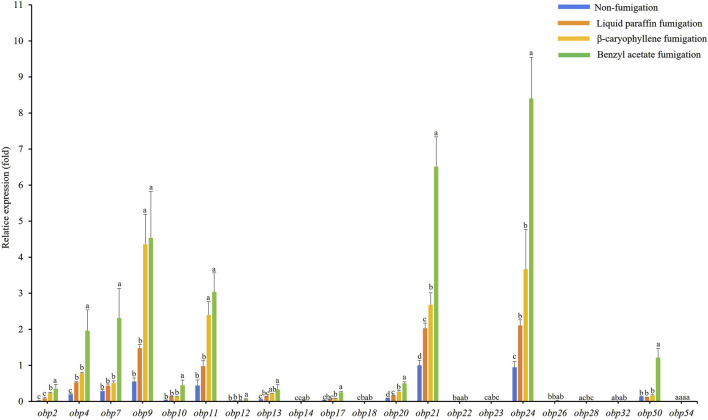
Effect of exposure to plant volatiles on expression levels of *OBP* genes in the antennae of *A*. *dissimilis* male adults Data are presented as mean ± SE. Different lowercase letters above the bars indicate significant difference between different treatments of the same gene (*P* < 0.05).

The expression levels of *AdisOBP11* were the highest in the female antennae, followed by *AdisOBP24*, *AdisOBP9*, *AdisOBP21*, and *AdisOBP4* (Figure 3). However, among the male antennae, *AdisOBP21* and *AdisOBP24* exhibited the highest gene expression, followed by *AdisOBP9*, *AdisOBP11*, *AdisOBP7*, *AdisOBP4*, *AdisOBP50*, *AdisOBP20*, *AdisOBP13*, and *AdisOBP10* in non-fumigation test (Figure 4). After fumigation, the expression of *AdisOBPs* in both female and male antennae showed varying degrees of increase compared with non-fumigation. The expression levels of *AdisOBP9* and *AdisOBP11* in the female antennae were significantly higher than those in the paraffin control after fumigation with β-caryophyllene. Conversely, the expression of *AdisOBP11*, *AdisOBP21*, and *AdisOBP24* in the female antennae after fumigation with benzyl acetate was significantly lower than that in the paraffin control (Figure 3). After fumigation with by β-caryophyllene, the expression of *AdisOBP9*, *AdisOBP11*, *AdisOBP24*, and *AdisOBP21* in the male antennae increased significantly compared with that in the paraffin control. After fumigation with benzyl acetate, the expression levels of *AdisOBP24*, *AdisOBP21*, *AdisOBP9*, *AdisOBP11*, *AdisOBP7*, *AdisOBP4*, and *AdisOBP50* in the male antennae were significantly higher than those in the paraffin control (Figure 4). After odor fumigation, the number of genes upregulated by *AdisOBP* in male antennae was greater than that in female antennae.

### 3.4 Modeling and molecular docking

**FIGURE 5:**
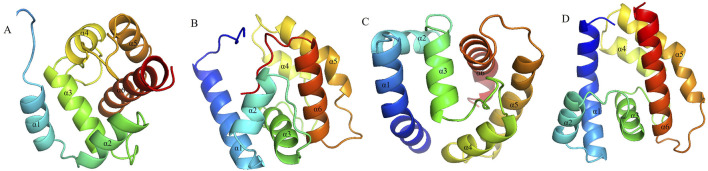
Structural modeling of OBP proteins in **(A)**. *dissimilis*. **(A)** AdisOBP9; **(B)** AdisOBP11; **(C)** AdisOBP21; **(D)** AdisOBP24.

**FIGURE 6:**
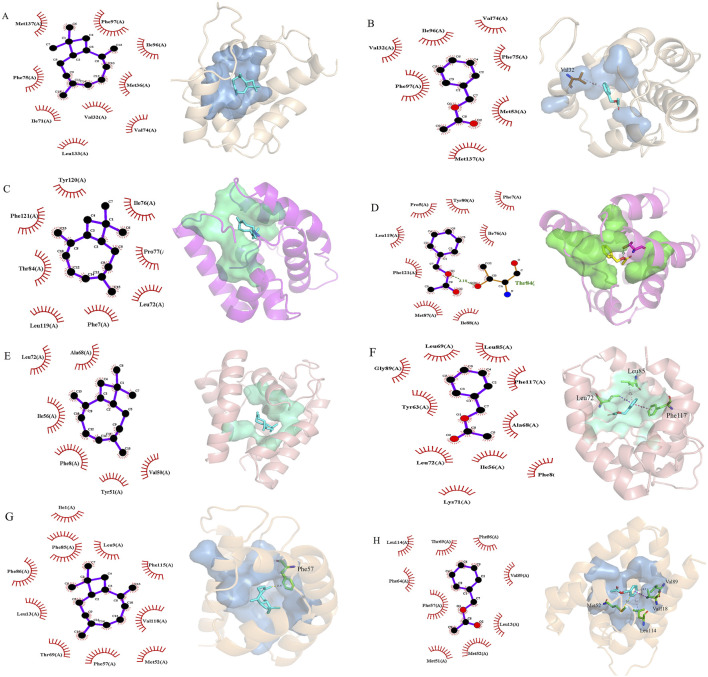
Molecular docking of AdisOBPs with volatile complexes. **(A)** AdisOBP9−β-caryophyllene; **(B)** AdisOBP9−benzyl acetate; **(C)** AdisOBP11−β-caryophyllene; **(D)** AdisOBP11−benzyl acetate; **(E)** AdisOBP21−β-caryophyllene; **(F)** AdisOBP21−benzyl acetate; **(G)** AdisOBP24−β-caryophyllene; **(H)** AdisOBP24−benzyl acetate. The left side shows a two-dimensional (2D) plane view and the right side shows a 3D view. The cyan green dotted line represents hydrogen bonds; the purplish red represents pi-pi stacking, and the blue-purple represents pi-sigma interaction.

Based on the results of the fumigation test, we selected AdisOBP9, AdisOBP11, AdisOBP21, and AdisOBP24, which exhibited significantly increased odor-induced expression, for homology modeling and molecular docking studies. Homologous modeling of AdisOBP9, AdisOBP11, AdisOBP21, and AdisOBP24 was respectively compared with the sequences of *Helicoverpa armigera* OBP3, *Pieris rapae* OBP10, *Agrotis ypsilon* OBP2, and *A*. *lepigone* OBP24. Sequence identities with the template were 76.19, 72.95, 72.80, and 100%, respectively. The stereochemical properties of the protein model were evaluated using the ramachandran plot. 93.10% of the amino acid residues in AdisOBP9 were located in the optimal region, 97.50% in AdisOBP11, 99.19% in AdisOBP21, and 99.20% in AdisOBP24 (Supplementary Figure S3). The model indicated that the four AdisOBP proteins share a similar structure with other insect OBPs and are characterized by six α-helical components (Figure 5). Odor ligands, including β-caryophyllene and benzyl acetate, were downloaded from the ZINC database, energy-minimized, and subsequently docked with AdisOBP9, AdisOBP11, AdisOBP21, and AdisOBP24. Molecular docking results suggest that potential hydrophobic and electrostatic interactions play significant roles in binding (Figure 6). The predicted free binding energies of AdisOBP9, AdisOBP11, AdisOBP21, and AdisOBP24 with β-caryophyllene were – 5.9, −7.6, −7.4, and −7.3 kJ/mol, respectively. The predicted free binding energies of the recombinant proteins AdisOBP9, AdisOBP11, AdisOBP21, and AdisOBP24 with benzyl acetate were – 4.3, −5.9, −5.7, and −5.7 kJ/mol, respectively (Supplementary Table S3). The Thr84 residue of AdisOBP11 was involved in the formation of hydrogen bonds with benzyl acetate. Hydrophobic interactions were observed between all compounds and AdisOBPs (Figure 6).

## 4 Discussion

Insect EAG technology enables the direct recording of the electrophysiological responses of insects to volatile chemical signals through a potential signal amplification system (Yan, 2011; Liu Y. et al., 2022; Lu et al., 2025). In this study, we recorded significant sex differences in the EAG responses of adults to 39 of 61 host plant volatile compounds. The EAG responses of females to 8 compounds were significantly higher than those of males. Conversely, the EAG responses to the remaining 31 volatile compounds were significantly lower in females than in males. These findings suggest that males in *A. dissimilis* adults are more sensitive to stimuli from the host plant volatiles. In general, insects, particularly female insects, rely more on volatiles released by host plants to locate optimal vegetative and oviposition sites for egg development and the growth of offspring larvae (or nymphs). Female insects often exhibit stronger and more sensitive EAG responses to host plant volatiles (Sun et al., 2014). For instance, *Phthorimaea operculella* (Das et al., 2007) and *Plutella xylostella* (Wu et al., 2020). It has also been reported that the EAG response in male adults is significantly higher than that in female adults. For example, *Epiphyas postvittana* (Suckling et al., 1996), *Cydia pomonella* (Casado et al., 2006), *Iragoides fasciata* (Huang et al., 2012), *Ostrinia furnacalis* (Xie et al., 2015), and *Adoxophyes orana* (Li et al., 2022). The reason for this phenomenon might be that male adults need to utilize plant volatiles to increase their response to their sex pheromones (Deng et al., 2004; Wei et al., 2013; Borrero-Echeverry et al., 2018; Jarrett and Miller, 2023). Therefore, when developing a plant-derived attractant, gender should be fully considered.

The reactions of EAG with various types of volatiles differ among *A. dissimilis* adults, even among compounds of the same type, there are significant differences. Compound functional groups are one of the reasons for the differences in the reaction intensity of adults to different types of volatile EAG response. Among the volatile components that enable insects to produce EAG reactions, we found that the relative EAG response of males to *trans*-2-hexenal, *cis*-3-hexen-1-ol, *trans*-2-Hexen-1-ol, and (*E*, *E*)-2,4-hexadienal were the highest, all greater than 1 mV, and these compounds all contained at least one carbon-carbon double bond. These results are similar to those of *Apanteles angaleti* (Trang and Dey, 2010), *Adoxophyes orana* (Li et al., 2022), and *Batocera horsfieldi* (Zhou et al., 2022), which indicating that pests may recognize different functional groups of volatile components in some degree.

In addition, the length of the carbon chain of compounds is also a factor that affects the strength of the EAG response of adults to similar volatile substances (Thode et al., 2008; Gaubert et al., 2020). Our results also confirm this phenomenon. For instance, with the growth of the carbon chains of these ketone compounds (acetylacetone, 5; 2-heptanone, 7; 2-nonanone, 9; *cis*-jasmone, 11; and β-lonone, 13), the EAG response caused by male adults also gradually intensifies (0.72 ± 0.07 mV, 0.50 ± 0.04 mV, 0.44 ± 0.02 mV, 0.27 ± 0.02 mV, and 0.19 ± 0.03 mV). Similar results have also been found in *Adoxophyes orana* (Li et al., 2022).

The same host-plant volatile components may have different conformations, such as isomers. Generally, insects have diverse EAG reactions to different isomers, as seen in *Monochamus alternatus* (Ren, 2014) and *Xylotrechus rusticus* (L.) (Yan et al., 2006). In this study, two isomers, *trans*-2-hexen-1-ol and *cis*-2-hexen-1-ol were detected. *Athetis dissimilis* male adults had a low EAG response to *cis*-2-hexen-1-ol but showed a high response to trans-2-hexen-1-ol. It indicated the chemical structure of volatile components may affect the EAG reactions of insects (Blažytė-Čereškienė et al., 2016; Lu et al., 2017).

The EAG response of female and male adults *A. dissimilis* to the volatile compounds displayed differences with varying concentrations and also among specific compounds at a given concentration. However, there were different critical values for the female and male antennal responses of *A. dissimilis* to different volatiles. When the concentration is lower or higher than this critical value, the neuronal response intensity of *A. dissimilis* may reach saturation or even reverse and decrease, manifested as the EAG response value no longer continuing to increase or beginning to decrease. This phenomenon indicates that the olfactory sensitivity of the *A. dissimilis* to different components of the host’s volatile substances varies, and similar results are also found in other herbivorous insects (Li et al., 2022; Luo et al., 2023). Therefore, in the development of plant-derived attractants, the selection of effective host plant volatile concentrations will be considered as a key issue limiting the preparation attractants.

Exposure to volatile compounds has been shown to alter the number of transcripts of the corresponding olfactory receptors involved in the detection of tested odors (von der Weid et al., 2015; Yin et al., 2019; Qian et al., 2024; Zhao et al., 2024). Therefore, we hypothesized that exposing adults to plant volatiles may help to identify the *OBP* genes responsible for sensing these odors. Our result found that the expression levels of *AdisOBP9* and *AdisOBP11* were significantly increased in female and male antennae exposed to β-caryophyllene. After fumigation with benzyl acetate, the expression levels of *AdisOBPs* in female antennae did not increase, however, the expression levels of *AdisOBP24*, *AdisOBP21*, *AdisOBP9*, *AdisOBP11*, *AdisOBP7*, *AdisOBP4*, and *AdisOBP50* in the male antennae were significantly higher. Similar findings have been reported previously. For instance, after 24 h of exposure to (*E*)-2-hexenol, the expression levels of *HoblOBP13*, *HoblOBP9*, and *HoblOBP4* in female beetles of *Holotrichia oblita* increased significantly compared with those in the control groups (Yin et al., 2019). Similarly, following chemical fumigation with *Oides leucomelaena*, the expression levels of *OleuOBP3*, *OleuOBP*5 and *OleuOBP*6 were significantly elevated compared with those in the control groups (Zhao et al., 2024). After fumigation with chemical compounds, the number of genes upregulated by AdisOBP in males was greater than that in females, which was the same as the result of the EAG test, that is, males were more sensitive to odors than females. The reason for this phenomenon might be that the male adults of *A. dissimilis* need more OBPs to recognize and combine the volatile substances of different host plants in order to improve the recognition of sex pheromones.

Many of the traps containing benzyl acetate components captured moths (Landolt et al., 2001; Robert and Meagher, 2002; Landolt et al., 2006), but the effect was not good when using benzyl acetate alone (Landolt et al., 2006; Szanyi et al., 2022). β-caryophyllene is a volatile substance found in various plants, and it can enhance the mating efficiency of male adults (Ul Haq et al., 2024). It has also been discovered that a mixture composed of β-caryophyllene and other compounds can significantly attract female adults, and can thus be used for the management and monitoring of female adults (Gharaei et al., 2020). Therefore, when using plant attractants for field control of the *A. dissimilis*, it is essential to take into account the synergistic effect of plant volatile compounds.

The 3D models demonstrated that the four AdisOBP proteins share a structure similar to that of other insect OBPs, featuring six α-helical components and a hydrophobic pocket (Pei et al., 2024; Yi et al., 2024; Yang et al., 2025). The hydrophobic interactions of OBPs play a crucial role in ligand binding, independent of the strict geometric constraints associated with intermolecular interactions such as hydrogen bonding (Wogulis et al., 2006; Scott et al., 2009; Li et al., 2019). Molecular docking results indicated that the binding energies of the four AdisOBP proteins with β-caryophyllene ranged from −5.9 to −7.6 kJ/mol, whereas those with benzyl acetate ranged from −4.3 to −5.9 kJ/mol. The binding capacity aligns with the findings of previous studies (Li et al., 2021). The binding energy of AdisOBP9 with Benzyl acetate is only −4.3 kJ/mol, and the binding energy is relatively weak. There are many reasons for this phenomenon. Sometimes the results of behavioral experiments do not match those of molecular experiments. The Thr84 amino acid residue in AdisOBP11 was involved in the formation of hydrogen bonds with benzyl acetate, suggesting that it actively participates in the binding to AdisOBP11. These findings provide valuable insights into the potential protein-binding sites necessary for protein-ligand interactions, which also represent effective targets for the development of new pest control techniques and management strategies (Deng et al., 2004; Wei et al., 2013; Borrero-Echeverry et al., 2018; Jarrett and Miller, 2023).

This study found that male adults exhibited greater sensitivity to host plant volatiles than in female adults. Male adults demonstrated higher EAG responses to aldehydes and alcohol volatiles, followed by esters, ketones, and thiazole volatiles. The results of the fumigation tests also indicated that a greater number of *AdisOBP* genes were upregulated in male adults than in female adults. Specifically, *AdisOBP9*, *AdisOBP11*, *AdisOBP21*, and *AdisOBP24* might be crucial genes involved in the recognition and transport of odorous molecules from the external environment. The key amino acid residues identified in the molecular docking results provided potential targets for future gene mutations and the development of new control strategies.

## Data Availability

The original contributions presented in the study are included in the article/Supplementary Material, further inquiries can be directed to the corresponding author.
