# Supplementary material for: Electroantennographic response and odorant-binding protein expression alterations induced by host plant volatiles in Athetis dissimilis (Hampson)
Source: Front Physiol. 2025 Jul 9;16:1619418. doi: 10.3389/fphys.2025.1619418 (PMC12283724; doi:10.3389/fphys.2025.1619418)
Supplement: Supplementary file 1 [file Supplementaryfile1.docx]

**Electroantennographic response and odorant-binding proteins expression** **alteration induced by host plant volatiles in** ***Athetis dissimilis* (Hampson)**

**Supplementary information**

**Table 1S:** Information of the 61 host-plant volatile compounds tested

| Tested compounds | Molecular formula | Purity | Cas no. | Source | Reference to plant sources |
| --- | --- | --- | --- | --- | --- |
| *Cis*-3-Hexenyl acetate | C_8_H_14_O_2_ | ≥ 98% | 3681-71-8 | Sigma-Aldrich | corn, wheat |
| Methyl jasmonate | C_13_H_20_O_3_ | ≥ 98% | 39924-52-2 | Sigma-Aldrich | corn, wheat |
| Benzyl acetate | C₉HO₂ | ≥ 99% | 140-11-4 | Sigma-Aldrich | corn, wheat |
| *Cis*-3-Hexenyl butyrate | C_10_H_18_O_2_ | ≥ 95% | 16491-36-4 | Sigma-Aldrich | corn, wheat |
| Geraniol | C_10_H_18_O | ≥ 97% | 106-24-1 | Sigma-Aldrich | corn, wheat |
| 1-Heptanol | C_7_H_16_O | ≥ 98% | 111-70-6 | Alfa | corn, wheat |
| Hexyl alcohol | C₆HO | ≥ 98% | 111-27-3 | Alfa | corn, wheat |
| Nerolidol | C_15_H_26_O | 98% | 7212-44-4 | Macklin | corn, wheat |
| *Trans*-2-hexen-1-ol | C_6_H_12_O | ≥ 96% | 928-95-0 | Macklin | corn, wheat |
| *Cis*-2-hexen-1-ol | C_6_H_12_O | 95% | 928-94-9 | Macklin | corn, wheat |
| *Cis*-3-hexen-1-ol | C_6_H_l2_O | ≥ 98% | 928-96-1 | Sigma-Aldrich | corn, wheat |
| Linalool | C_10_H_18_O | ≥ 97% | 78-70-6 | Sigma-Aldrich | corn, wheat |
| 1-Octen-3-ol | C_8_H_16_O | ≥ 98% | 3391-86-4 | Sigma-Aldrich | corn, wheat |
| 1-Nonanol | C_9_H_20_O | 98% | 143-08-8 | Sigma-Aldrich | corn, wheat |
| Octadecanol | C_18_H_38_O | ≥ 99% | 112-92-5 | Sigma-Aldrich | corn, wheat |
| *Trans*-Nerolidol | C_15_H_26_O | ≥ 95% | 40716-66-3 | Sigma-Aldrich | corn |
| 2-Heptanone | C_7_H_14_O | ≥ 98% | 110-43-0 | Sigma-Aldrich | corn, wheat |
| Camphor | C_10_H_16_O | 96% | 76-22-2 | Sigma-Aldrich | corn, wheat |
| 2-Nonanone | C_9_H_18_O | ≥ 99% | 821-55-6 | Sigma-Aldrich | corn, wheat |
| *Cis*-Jasmone | C_11_H_16_O | ≥99.37% | 488-10-8 | Sigma-Aldrich | corn, wheat |
| β-Ionone | C_13_H_20_O | ≥ 97% | 79-77-6 | Sigma-Aldrich | corn, wheat |
| β-pinene | C_10_H_16_ | ≥ 97% | 127-91-3 | Sigma-Aldrich | corn, wheat |
| Myrcene | C_10_H_16_ | ≥ 90% | 123-35-3 | Sigma-Aldrich | corn |
| β-caryophyllene | C_15_H_24_ | ≥ 80% | 87-44-5 | Sigma-Aldrich | corn, wheat |
| Camphene | C_10_H_16_ | 95% | 79-92-5 | Sigma-Aldrich | corn |
| (*R*)-(+)-Limonene | C_10_H_16_ | 97% | 5989-27-5 | Sigma-Aldrich | corn, wheat |
| (*S*)-(-)-Limonene | C_10_H_16_ | ≥ 95% | 5989-54-8 | Sigma-Aldrich | corn, wheat |
| Limonene | C_15_H_24_ | 95% | 18794-84-8 | Macklin | corn, wheat |
| Tridecane | C_13_H_28_ | 98% | 629-50-5 | Macklin | corn, wheat |
| Undecane | C_11_H_24_ | ≥ 99% | 1120-21-4 | Sigma-Aldrich | corn, wheat |
| Hexadecene | C_16_H_32_ | ≥ 99% | 544-76-3 | Sigma-Aldrich | corn, wheat |
| Heptadecane | C_17_H_36_ | 99% | 629-78-7 | Sigma-Aldrich | corn, wheat |
| Tricosane | C_23_H_48_ | 99% | 638-67-5 | Sigma-Aldrich | corn, wheat |
| 4-Ethylbenzaldehyde | C_9_H_10_O | 98% | 4748-78-1 | Sigma-Aldrich | corn, wheat |
| Decanal | C_10_H_20_O | ≥ 97% | 112-31-2 | Sigma-Aldrich | corn |
| *Trans*-2-Hexenal | C_6_H_10_O | 98% | 6728-26-3 | Sigma-Aldrich | corn, wheat |
| Octanal | C_8_H_16_O | ≥ 97% | 124-13-0 | Sigma-Aldrich | wheat |
| Benzaldehyde | C₇H₆O | ≥ 98% | 100-52-7 | Sigma-Aldrich | wheat |
| Dodecyl aldehyde | C_₁₂_H_₂₄_O | ≥ 98% | 112-54-9 | Sigma-Aldrich | corn, wheat |
| Undecanal | C_11_H_22_O | ≥ 97% | 112-44-7 | Macklin | corn |
| Nonanal | C_9_H_18_O | ≥ 95% | 124-19-6 | Sigma-Aldrich | corn |
| Hexanal | C₆H₁₂O | ≥ 97% | 66-25-1 | Sigma-Aldrich | corn, wheat |
| p-Anisaldehyde | C_8_H_8_O_2_ | ≥ 97.5% | 123-11-5 | Sigma-Aldrich | corn, wheat |
| Indole | C₈H₇N | ≥ 98% | 120-72-9 | Sigma-Aldrich | corn |
| Lauric acid | C_12_H_24_O_2_ | ≥ 98% | 143-07-7 | Sigma-Aldrich | corn, wheat |
| (*E*, *E*)-2,4-Hexadienal | C_6_H_8_O | 95% | 142-83-6 | Macklin | wheat |
| Styrene | C_8_H_8_ | ≥ 99% | 100-42-5 | Sigma-Aldrich | corn, wheat |
| Geranyl acetate | C_12_H_20_O_2_ | ≥ 97% | 105-87-3 | Sigma-Aldrich | corn, wheat |
| Methyl salicylate | C_8_H_8_O_3_ | ≥ 99% | 119-36-8 | Sigma-Aldrich | corn |
| Methyl benzoate | C_8_H_8_O_2_ | ≥ 98% | 93-58-3 | Sigma-Aldrich | corn, wheat |
| Hexyl acetate | C_8_H_16_O_2_ | ≥ 98% | 142-92-7 | Sigma-Aldrich | corn, wheat |
| 2-Ethylhexyl acetate | C_10_H_20_O_2_ | ≥ 99% | 103-09-3 | Sigma-Aldrich | wheat |
| 1,4-Butyrolactone | C_4_H_6_O_2_ | ≥ 99% | 96-48-0 | Macklin | corn, wheat |
| Benzothiazole | C₇H₅NS | ≥ 96% | 95-16-9 | Sigma-Aldrich | corn |
| Octadecane | C_18_H_38_ | 99% | 593-45-3 | Sigma-Aldrich | corn, wheat |
| Eicosane | C₂₀H₄₂ | 99% | 112-95-8 | Sigma-Aldrich | corn, wheat |
| Pentadecane | C_15_H_32_ | ≥ 98% | 629-62-9 | Sigma-Aldrich | corn, wheat |
| Dodecane | C_12_H_26_ | ≥ 99% | 112-40-3 | Sigma-Aldrich | wheat |
| p-Xylene | C_8_H_10_ | ≥ 99% | 106-42-3 | Sigma-Aldrich | wheat |
| Acetylacetone | C₅H₈O₂ | ≥ 99.5% | 123-54-6 | Sigma-Aldrich | wheat |

**Table S2:** Primers used for expression analysis by qRT-PCR in this study.

| Genbank Accession No. | Primer name | Sequence（5’-3’） |
| --- | --- | --- |
| KT361883 | AdisGAPDH-F | CTGCTCATTTAGAGGGTGGTGC |
|  | AdisGAPDH-R | TCTTCTGGGTAGCGGTGGTAG |
| MH900291 | AdisOBP2-F | TACTCCTCACGCTCCTACCT |
|  | AdisOBP2-R | AGCAACAGCCACTTCATCTTC |
| KT220704 | AdisOBP4-F | CTTACGCGATGACAAGACAACAAC |
|  | AdisOBP4-R | CCGCTGGAAACATGACATCTACTTGT |
| KT220705 | AdisOBP7-F | GATGTGACTGAAGATCAAGTGGG |
|  | AdisOBP7-R | ATCCGGTGGATACATGAGGTC |
| MH900295 | AdisOBP9-F | GAACGGAACGGAAAGACCAT |
|  | AdisOBP9-R | ACATCAGGCATCTCAGGAATTG |
| KT220706 | AdisOBP10-F | TCCTGTTACGGATGATCAAGTTG |
|  | AdisOBP10-R | GGCTATGAATGCACAAAGATACAGC |
| MH900296 | AdisOBP11-F | GACGACGATGTTGTGGACTAC |
|  | AdisOBP11-R | ATCTGTGTACTTCAAATGCTCTCTG |
| KT220703 | AdisOBP12-F | TCAGCGAAGAATGGCTTAACAGAAG |
|  | AdisOBP12-R | TAACAGGAACCTTCATTTCTGTCG |
| MH900297 | AdisOBP13-F | ACGGAGACCAAGGTTGATGA |
|  | AdisOBP13-R | GCAGAGGGCATACTTCTTTAGC |
| MH900298 | AdisOBP14-F | CACCAGATGTGATCTACAGTTGTC |
|  | AdisOBP14-R | CAGCCTGTCGCACTCATTAG |
| MH900301 | AdisOBP17-F | ACGGTGCTCAATGCTTGTTAT |
|  | AdisOBP17-R | CCAATACTGCTATGCCCTTCTG |
| MH900302 | AdisOBP18-F | AAGGATTCCGAGGCACTGTT |
|  | AdisOBP18-R | CCGCAACTCTCCATAACTTCATC |
| MH900304 | AdisOBP20-F | ATCGTGTCAACCAAAGAATAATGTC |
|  | AdisOBP20-R | GCCATGTAGCACTTCACTTCT |
| MH900305 | AdisOBP21-F | CAGCCATCAAGCCGATCATAG |
|  | AdisOBP21-R | TTGTAGACACAGCCGAGGAA |
| MH900306 | AdisOBP22-F | CATAGTGGCGTGCTCTCAAG |
|  | AdisOBP22-R | TGAAGACACAGGCAATGAAACA |
| MH900307 | AdisOBP23-F | GTCTTGCTTCTTAGGTTGTGTCAT |
|  | AdisOBP23-R | GTCGTCATCGCTGCTTACATAC |
| MH900308 | AdisOBP24-F | AGAAGACCGTTTCATTCGCTAAG |
|  | AdisOBP24-R | GACACATTCCTCTCCAACAGTT |
| MH900310 | AdisOBP26-F | GTGGAGATGGACGAGGACAT |
|  | AdisOBP26-R | GCACTTGATGTAGCACTTGAGC |
| MH900312 | AdisOBP28-F | GAGAGATCGGGAGGGAGAAAT |
|  | AdisOBP28-R | GTCGCTTCCATATTCGTCACTT |
| MH900316 | AdisOBP32-F | AATTGGTGGACTCCTGTGTTG |
|  | AdisOBP32-R | GTTCTATCTTCTGTGGTGCTGTG |
| MH900334 | AdisOBP50-F | TGGTGAAGTTTAGTGTTGTGTGTT |
|  | AdisOBP50-R | CGGCGATAGGTGTTATTGCTT |
| MH900338 | AdisOBP54-F | GAAGCAGATTGGCGTTATGGA |
|  | AdisOBP54-R | CCGTTAATATGTGAGCAGGAATGA |

**Table S3:** Molecular docking of OBPs of *Athetis dissimilis* to plant volatiles

| Recombinant protein | | Ligand | |
| --- | --- | --- | --- |
|  |  | β-caryophyllene | Benzyl acetate |
| OBP9 | Binding Energy (kJ/mol) | -5.9 | -4.3 |
|  | Hydrophobic Interactions | Val32, Met36, Ile71, Val74, Phe75, Ile96, Phe97, Leu133, Met137 | Val32, Met53, Val74, Phe75, Ile96, Phe97, Met137 |
| OBP11 | Binding Energy (kJ/mol) | -7.6 | -5.9 |
|  | Hydrophobic Interactions | Phe7, Leu72, Ile76, Pro77, Thr84, Leu119, Tyr120, Phe121 | Pro5, Phe7, Ile76, Tyr80, Met87, Ile88, Leu119, Phe121 |
| OBP21 | Binding Energy (kJ/mol) | -7.4 | -5.7 |
|  | Hydrophobic Interactions | Phe8, Val50, Ile56, Ala68, Leu72, Tyr51 | Leu69, Leu85, Phe117, Gly89, Tyr63, Ala68, Phe8, Ile56, Leu72, Lys71 |
| OBP24 | Binding Energy (kJ/mol) | -7.3 | -5.7 |
|  | Hydrophobic Interactions | Ile1, Leu9, Leu13, Phe15, Met52, Phe57, Thr69, Phe85, Phe86, Phe115, Val118 | Leu13, Met51, Met52, Phe57, Phe64, Thr69, Phe86, Val89, Leu14 |

**Table S4:** Analysis of MolProbity score, Ramachandran favored and QMEAN in OBPs

| Protein name | MolProbity score | Ramachandran favored | QMEAN |
| --- | --- | --- | --- |
| OBP9 | 0.94 | 93.10% | -1.47 |
| OBP11 | 0.78 | 97.5% | 0.06 |
| OBP21 | 0.70 | 99.19% | 0.69 |
| OBP24 | 0.50 | 99.20% | 1.99 |


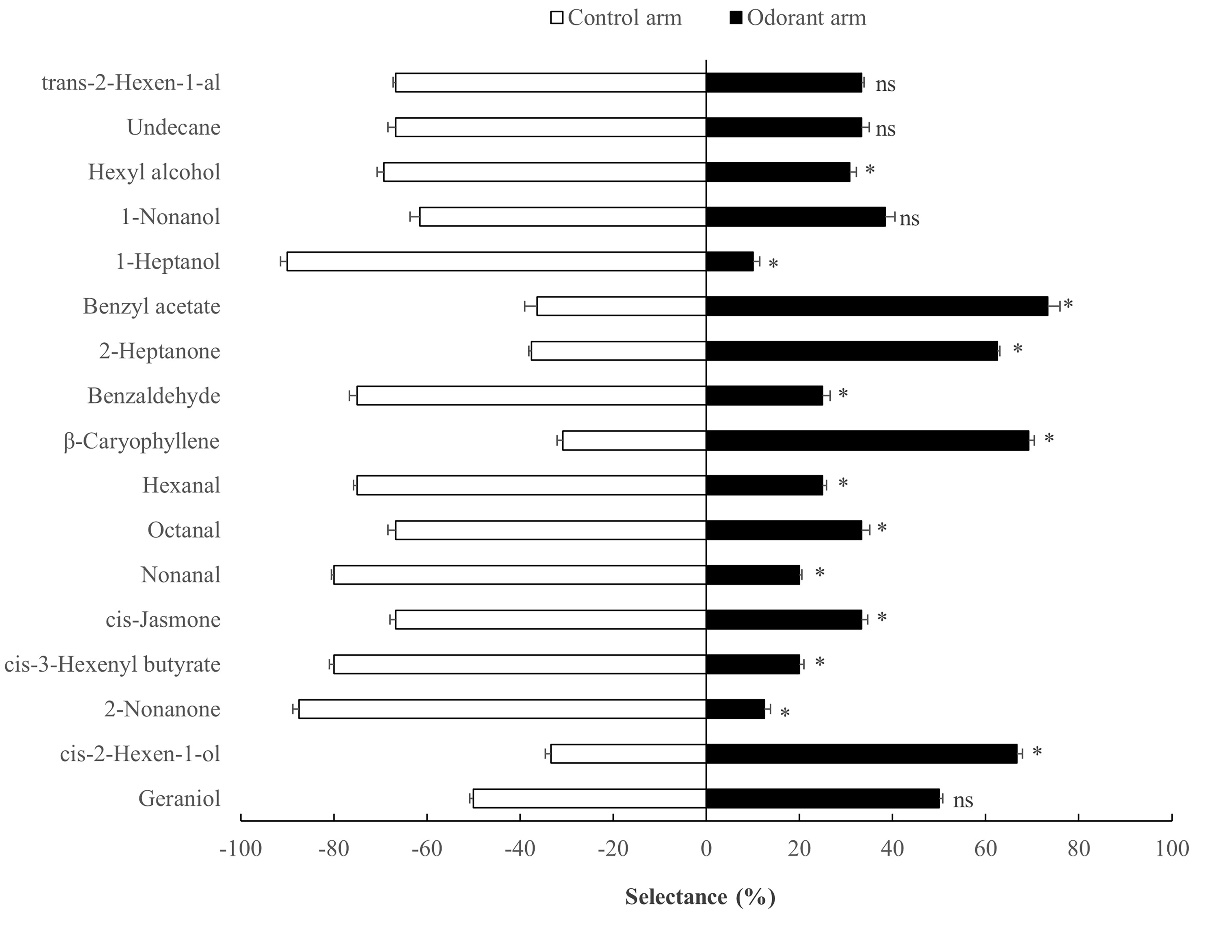


Figure S1: Behavioral responses of *Athetis dissimilis* female adult to plant volatile in a Y-tube olfactometer.

The concentration of the test compound is 1 M; All the data were subjected to chi-square test, *P* < 0.05; The asterisk (*) indicates significant differences, while NS indicates no significant differences.


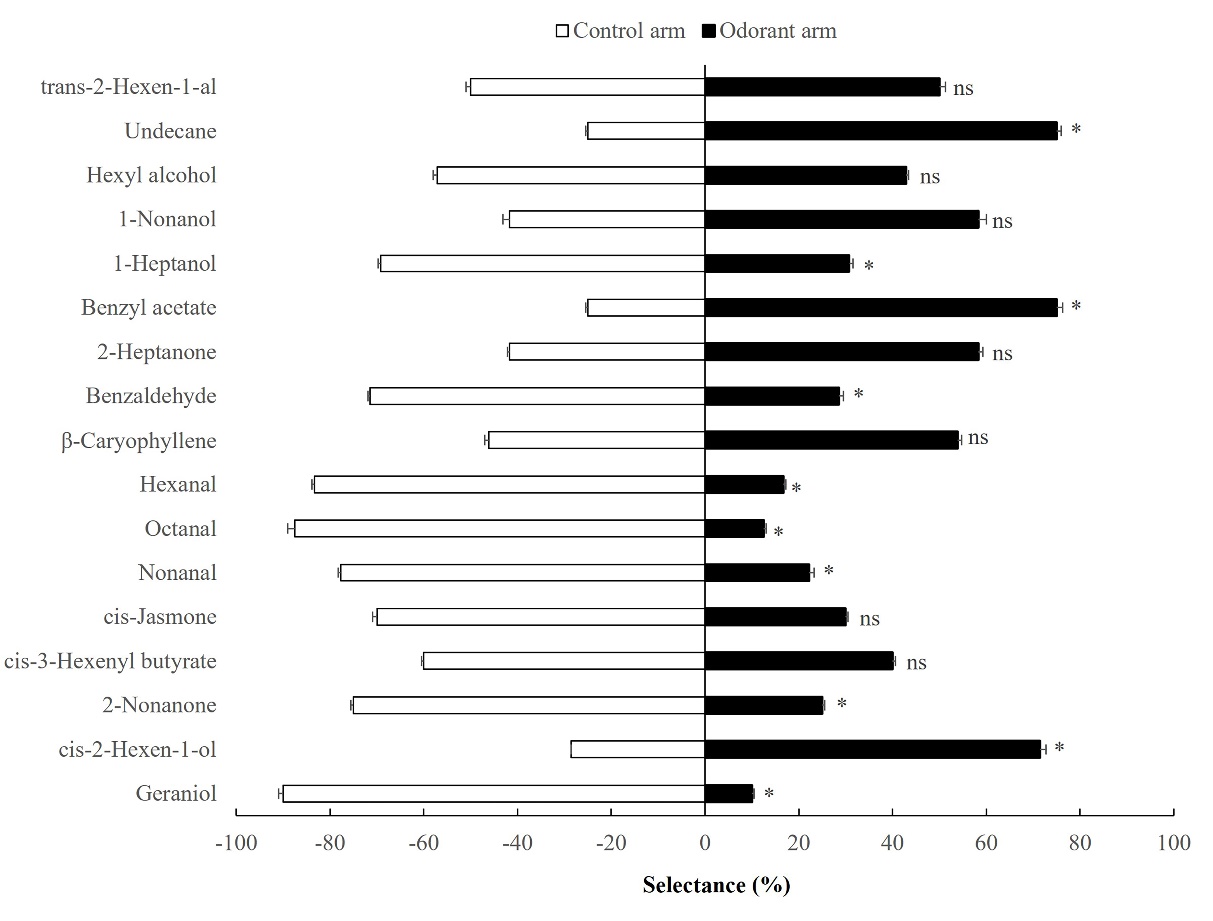


Figure S2: Behavioral responses of *Athetis dissimilis* male adult to plant volatile in a Y-tube olfactometer.

The concentration of the test compound is 1 M. All the data were subjected to chi-square test, *P* < 0.05; The asterisk (*) indicates significant differences, while NS indicates no significant differences.


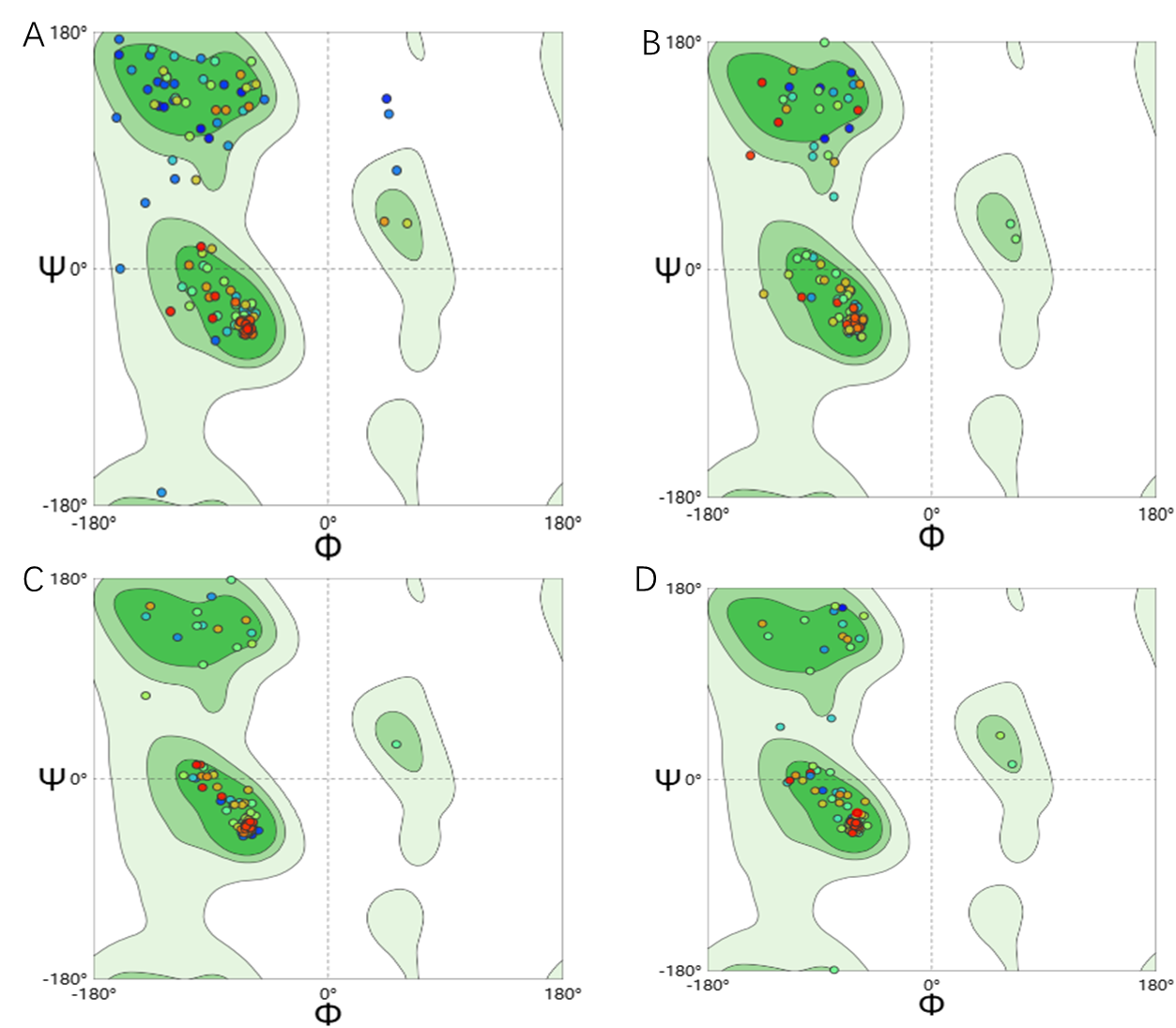


Figure S3: AdisOBPs Homologous Modeling Quality assessment.

A: AdisOBP9; B: AdisOBP11; C: AdisOBP9; D: AdisOBP9.
